# Cholesterol Depletion in Adipocytes Causes Caveolae Collapse Concomitant with Proteosomal Degradation of Cavin-2 in a Switch-Like Fashion

**DOI:** 10.1371/journal.pone.0034516

**Published:** 2012-04-06

**Authors:** Michael R. Breen, Marta Camps, Francisco Carvalho-Simoes, Antonio Zorzano, Paul F. Pilch

**Affiliations:** 1 Department of Biochemistry, Boston University School of Medicine, Boston, Massachusetts, United States of America; 2 Departament de Bioquímica i Biologia Molecular, Facultat de Biologia, Universitat de Barcelona, Barcelona, Spain; 3 CIBER de Diabetes y Enfermedades Metabólicas Asociadas (CIBERDEM), Instituto de Salud Carlos III, Barcelona, Spain; 4 IBUB Institute of Biomedicine of the University of Barcelona, Barcelona, Spain; 5 Institute for Research in Biomedicine (IRB Barcelona), Barcelona, Spain; 6 Department of Medicine, Boston University School of Medicine, Boston, Massachusetts, United States of America; The University of Queensland, Australia

## Abstract

Caveolae, little caves of cell surfaces, are enriched in cholesterol, a certain level of which is required for their structural integrity. Here we show in adipocytes that cavin-2, a peripheral membrane protein and one of 3 cavin isoforms present in caveolae from non-muscle tissue, is degraded upon cholesterol depletion in a rapid fashion resulting in collapse of caveolae. We exposed 3T3-L1 adipocytes to the cholesterol depleting agent methyl-β-cyclodextrin, which results in a sudden and extensive degradation of cavin-2 by the proteasome and a concomitant movement of cavin-1 from the plasma membrane to the cytosol along with loss of caveolae. The recovery of cavin-2 at the plasma membrane is cholesterol-dependent and is required for the return of cavin-1 from the cytosol to the cell surface and caveolae restoration. Expression of shRNA directed against cavin-2 also results in a cytosolic distribution of cavin-1 and loss of caveolae. Taken together, these data demonstrate that cavin-2 functions as a cholesterol responsive component of caveolae that is required for cavin-1 localization to the plasma membrane, and caveolae structural integrity.

## Introduction

Small (60–80 nm) invaginations of the cell surface called caveolae are common features of many cell types, which possess diverse physiological roles, for example, endothelial and epithelial cells, cardiac and skeletal muscle [1], [2], and of particular relevance herein, adipocytes [3], [4]. In line with this physiological diversity, caveolae have been found to play a role in numerous biological processes including signal transduction, endocytosis, mechano-transduction, cellular viral entry and regulation of fuel metabolism [1], [2], [3], [4]. Considering the pleiotropic nature of their possible physiological and functional roles, there has been no grand unified theory for the biochemical/mechanistic properties of caveolae, and most efforts to understand mechanism(s) have focused on the critical protein constituents of caveolae, the caveolins and cavins. The caveolins consist of three isoforms (Cav1-3) of small (151–178 amino acids) integral membrane proteins positioned entirely on the cytoplasmic face of the plasma membrane, Cav1 and -2 being expressed together in non-muscle tissue and Cav-3 being muscle specific [5]. There are four cavin isoforms ranging in size from 260 to 418 residues (murine) with the properties of peripheral membrane proteins, and they have leucine zipper (cavin-1-3) and PEST (proline, aspartate, serine, threonine) domains (all). Cavin-1 and cavin-2 are critical for caveola formation, whereas cavin-3 and cavin-4, the last being muscle-specific, may be dispensable in this regard [1], [2], [6]. The caveolins have been postulated to have a number of specific biochemical actions, but little is known in this regard concerning the cavins.

The physiological importance of caveolae has been underscored by the phenotypes of organisms lacking these structures as a result of natural mutations of caveolins and cavin-1 in humans and gene knockouts in mice. Loss of murine Cav1 causes vascular defects and insulin resistance [7], [8], [9], [10] and similar pathologies are seen in humans harboring Cav1 null alleles [11], [12]. Mice lacking Cav-3 have metabolic defects as well as muscular dystrophy [13], [14] as do humans with inactivating mutations in this protein [15]. Cavin-1 knockout mice exhibit insulin resistance and metabolic defects [16], a phenotype similar or identical to that of humans lacking this protein, who also have muscle and cardiac abnormalities [17], [18], [19], [20].

The insulin resistance metabolic phenotype of Cav1 and cavin-1 deficient mammals derives, at least in part, from defects in lipid storage in adipocytes, and includes diminished insulin action and abnormal lipolysis [4], [21], [22]. Although a complete molecular picture describing the properties of the caveola deficient fat cell is still lacking, an inability to store fat normally by whatever mechanism is associated with additional metabolic dysfunctions in other peripheral tissues, namely liver and muscle [23]. Because caveolae comprise as much as 50% of the plasma membrane area in primary fat cells [24], probably the highest level of any cell type, it is not surprising that their absence compromises adipocyte function. Moreover, the large lipid droplets, the triglyceride storage organelle of the fat cell [25] also serve as the largest reservoir of free cholesterol in the body [26], although the dynamics of this pool have not heretofore been investigated.

An early-recognized feature of caveolae is their dependence on cholesterol, loss of which causes these structures to lose their characteristic shape [27], [28]. Indeed, Cav1 was shown to bind cholesterol in a stoichiometric fashion [29]. These prior studies of the cholesterol-dependency of caveolae focused largely on the behavior of Cav1, the only protein known to be required for caveolae structure in non-muscle cells until the recent studies of the cavins, which documented their role in caveolae formation [16], [30], [31], [32]. We revisited the cholesterol depletion experimental paradigm in adipocytes in light of the possible role(s) of the cavin proteins in order to gain possible insight into cholesterol dynamics and the hierarchy and relationships amongst caveolae protein constituents in caveolae biology/biochemistry. Remarkably, we find that cavin-2 behaves in a switch-like fashion with regard to plasma membrane (PM) cholesterol content, and it is required for caveolae integrity and cavin-1 localization to the PM in adipocytes.

## Materials and Methods

### Antibodies

Polyclonal antibody directed against Cav1 was obtained from BD Transduction Laboratories (San Jose, CA, USA). Anti-actin and anti-GAPDH were purchased from Sigma (St. Louis, MO., USA). Polyclonal rabbit antibodies recognizing cavin-1, cavin-2, and cavin-3 were generated by 21st Century Biochemicals (Marlboro, MA, USA) using synthetic peptides as described [6]. Anti-rabbit Cy3 conjugated secondary antibody was from Jackson ImmunoResearch Laboratories, Inc. (West Grove, PA, USA).

### Reagents

TransIt-293 transfection reagent was purchased from Mirus (Madison, WI, USA). Simvastatin was purchased from Cayman Chemical (Ann Arbor, MI, USA). Methyl-β-cyclodextrin, filipin III, nystatin, cholesterol-loaded cyclodextrin, and chloroquine were purchased from Sigma (St. Louis, MO, USA) as were dexamethasone (DEX), 3-isobutyl-1-methylxanthine (MIX), Tetramethyl Rhodamine Isothiocyanate (TRITC)-Phalloidin and insulin. Lipoprotein-deficient fetal bovine serum (FBS) was purchased from Kalen Biomedical (Montgomery Village, MD, USA). MG-132 was purchased Calbiochem (Gibbstown, NJ, USA). Aprotinin, pepstatin and leupeptin were from American Bioanalytical, Natick, MA, USA.

### Cell culture

Primary mouse embryonic fibroblasts (MEFs) from wild type mice and MEFs from caveolin-1 null mice were kindly provided by Dr. Robert Parton, University of Queensland, Australia [33]. 3T3-L1 Fibroblasts (from ATCC) were maintained in DMEM containing 4.5 g/liter glucose and l-glutamine from Mediatech Inc., (Herndon, VA, USA) supplemented with 10% calf serum and 100 units/ml penicillin, and 100 µg/ml streptomycin (Invitrogen). 48 hours post-confluence, 3T3-L1 fibroblasts were induced to differentiate by changing the media to DMEM supplemented with 10% fetal bovine serum, 0.5 mm 3-isobutylmethylxanthine, 1 µM dexamethasone, and 1.7 µM insulin [34]. 48 hours post induction, the induction medium was removed and cells were maintained in DMEM with 10% fetal bovine serum. NIH-3T3 (from ATCC) and MEFs were maintained in DMEM with 10% fetal bovine serum in 5% CO_2_. Induction of differentiation for caveolin-1 null MEFs was identical to the protocol for 3T3-L1 cells except that Troglitazone (5 µM) (gift from Pfizer, Groton, CT, USA) was added through induction and differentiation.

### Lentiviral infection

HEK-293T (from Open Biosystems) cells were grown in Dulbecco's modified Eagle's medium (DMEM) containing 10% Fetal Bovine serum (Invitrogen), to 90% confluence in p100 dishes, at which stage they were dissociated with trypsin and plated into p150 dishes. After re-plating for 24 hours, the cells were transfected with 24 µg of backbone expressing shRNA against either eGFP or cavin-2, 1.2 µg of TAT, 1.2 µg of REV, 1.2 µg Gag/Pol, and 2.4 µg of Vsv-G. Two days post-transfection, medium was collected from the cells, and passed through a 0.45 µM filter with 8 µg/mL (final) polybrene onto target cells. Two days post infection, cells were selected with 2.5 µg/mL puromycin. Following selection, cells were screened for the efficiency of knockdown. The lentiviral vectors expressing shRNA against cavin-2 were purchased as a set from Open Biosystems (Hunstville, AL, USA).

### Preparation of Whole Cell Extracts

Cells were washed twice with PBS and incubated for 30 minutes on ice with cold RIPA buffer (50 mM Tris, pH 7.4; 150 mM NaCl; 1% Nonidet, 0.5% sodium deoxycholate and 0.1%SDS) with a protease inhibitor cocktail (aprotinin 10 µg/ml, pepstatin 1 µg/ml and leupeptin 1 µg/ml), or for some experiments, the same ingredients with SDS at 1%. Lysates were placed on an end-over-end rocker for 30 minutes at 4C and spun down for 10 min at 16,000× g at 4C. Protein concentrations were determined in the supernatant using BCA reagent (Pierce, Rockford, IL, USA).

### Cell Fractionation

Cells were incubated with or without the indicated treatment as labeled, and then washed twice with cold PBS, and one time with cold HES buffer prior to homogenization with a Teflon-glass tissue homogenizer in HES buffer. Homogenates were spun at 1000× g to remove nuclei and debris. Homogenates were then spun at 231,000× g to generate a pellet containing membrane fractions and a supernatant containing cytosol. Buffers used with fractionation contained a mix of protease inhibitors, which contained 1 µM aprotinin, 10 µM leupeptin, 1 µM pepstatin.

### Cholesterol determination

The assay was performed as outlined previously [35]. Briefly, the total amount of cellular cholesterol was determined by extracting lipids [36] from whole cell extracts and membrane preparations, and the lipid phase was assayed by a colorimetric procedure (horseradish peroxidase) to detect cholesterol.

### Gel Electrophoresis and Immunoblotting

Proteins were resolved by SDS-PAGE and electrophoretically transferred to PVDF membrane (Bio-Rad, Hercules, CA, USA). The membrane was blocked with 10% nonfat dry milk in PBS containing 0.5% Tween-20 for 1 hour at room temperature. Primary antibodies were detected using secondary antibodies conjugated to horseradish peroxidase (Sigma) and enhanced-chemiluminescence substrate from PerkinElmer Life Sciences (Boston, MA, USA). Quantitative analysis of Western blots was performed with software provided with a Fujifilm LAS4000 scanner used for the blots.

### Confocal Laser Scanning Microscopy

Cells were grown in p100 dishes and induced to differentiate as described. On day 6 of differentiation cells were trypsinized, and plated into 6 well plates containing coverslips coated with 0.1% gelatin (Millipore, Billerica, MA<USA) On day 8 experiments were conducted and cells were fixed with 4% paraformaldehyde in PBS for 15 min at room temperature. Cells were permeabilized with solution A (0.1% saponin (Sigma) and 0.4% BSA in PBS) for 10 min and then blocked for 1 hour at room temperature in 5% normal goat serum (Sigma) in PBS. Staining was performed with indicated antibodies overnight at 4°C at a dilution of 1/100 in 1% normal goat serum in PBS. Following staining cells were washed four times with buffer A. Cells were incubated anti-rabbit Cy3-conjugated secondary antibody at a dilution of 1/250 in 1% normal goat serum in PBS for 1 hour at room temperature. The cells were washed again for 3 times with solution A and then mounted with Vectashield mounting medium with DAPI (Vector Laboratories, Inc. Burlingame, CA, USA). The stained cells were observed using a Zeiss 510 confocal laser-scanning microscope (Carl Zeiss, Thornwood, NY, USA). Images were processed using LSM 510 Image software. Microscopy data shown (confocal and EM) are representative of 3 or more experiments as are all Western blot experiments.

### Electron Microscopy

Freeze drying of 3T3-L1 membrane lawns was performed as described [37], [38]. Briefly, plasma membranes lawns were fixed with glutaraldehyde, and after washing with water and methanol as cryoprotectant, they were rapidly frozen against a copper block cooled by liquid nitrogen. Plasma membranes were then freeze-dried in a freeze-etching unit, and rotatory replicas of platinum-carbon were obtained following the Heuser and Anderson technique [39].

## Results

### Methyl-β-cyclodextrin (MβCD) exposure in adipocytes causes loss of caveolae, cavin-2 degradation and redistribution of cavin-1 to the cytosol

**Figure 1:**
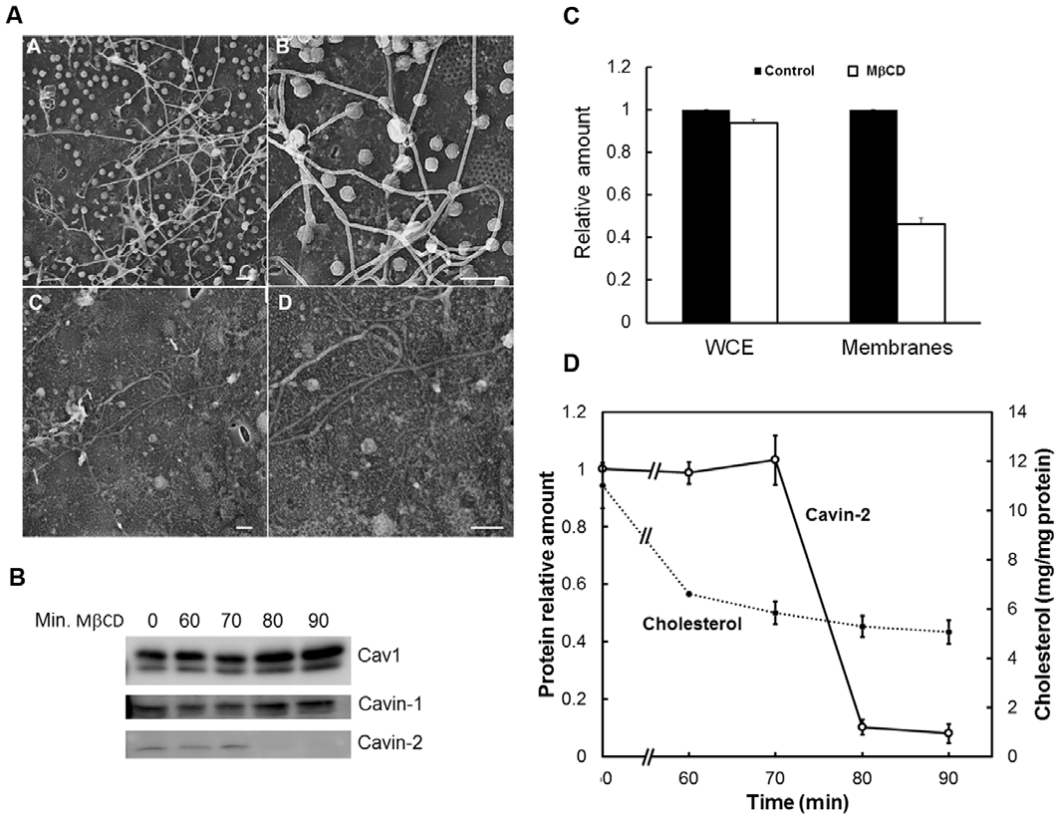
Cholesterol depletion collapses caveolae and causes loss of cavin-2 in a switch-like fashion (A). Freeze drying electron micrograph depicting the inner surface of the adipocyte plasma membrane before (panels A and B) and after (panels C and D) cholesterol depletion by 20 mM MβCD for 60 min. (left and right panels respectively, scale bar represents 200 nm). (**B**). 3T3-L1 adipocytes were treated with 20 mM MβCD for the indicated time points, lysates were prepared in RIPA buffer and analyzed by SDS-PAGE followed by Western blotting as described in Methods. (**C & D**). Cholesterol was determined as described in the Methods section for whole cell extracts and/or membranes (**C**, 90 min.) or at the times indicated (**D**) after MβCD exposure. The amount of cavin-2 was determined using a Fujifilm LAS-4000 Image Analyzer. The experiments of 1A, 1B and 1D are representative of >4 such experiments and C shows the mean ± S.E. for triplicate cell preparations. The value for cavin-2 at time zero was set to 1.0 and subsequent time points are expressed as ratios of this.

Cholesterol depletion/sequestration has been shown to disrupt caveolar structure [27], [28], [40], which we show for cultured murine adipocytes in Figure 1A by freeze drying electron microscopy (EM) of plasma membrane “lawns” [37] following cellular treatment with 20 mM MβCD. Moreover, as shown by Western blotting of cell lysates from fat cells exposed to MβCD for the times indicated (Figure 1B), there was no significant change in Cav1 or cavin-1 protein expression during the 90 minute time course, but strikingly, cavin-2 levels were reduced by 90% in the interval between 70 and 80 minutes of MβCD exposure. We measured total and plasma membrane (PM) cholesterol as a function of time of MβCD exposure and the former is unchanged (Figure 1C), but the latter (Figure 1D) shows that the loss of cavin-2 occurs upon a change in PM cholesterol from 0.53% to 0.48% of the control value. Thus, cavin-2 is highly sensitive to small changes over a narrow window of PM cholesterol levels (Fig. 1D).

**Figure 2:**
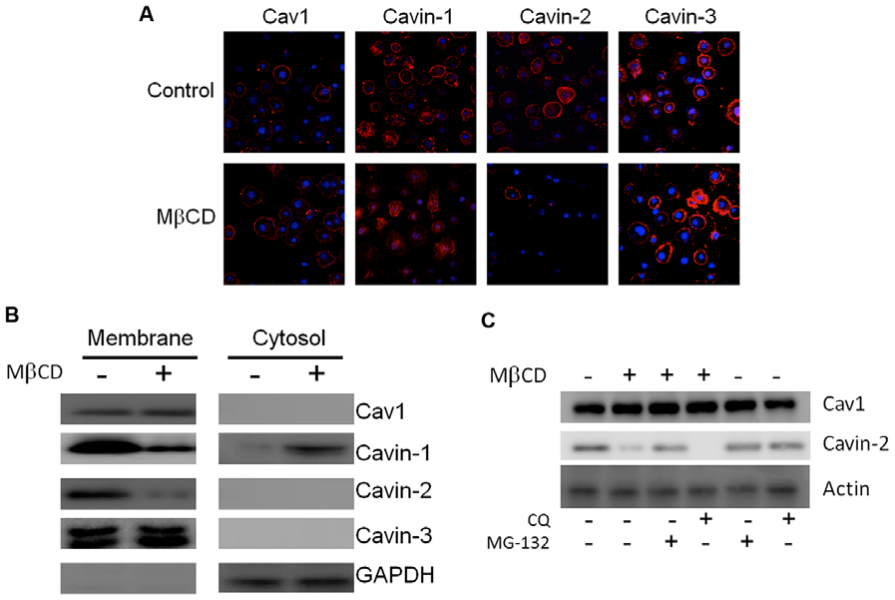
Cavin-2 is degraded by the proteosome and Cavin-1 redistributes to the cytosol following cholesterol depletion. 3T3-L1 adipocytes were treated with or without MβCD (20 mM, 90 minutes) and subjected to either (**A**) immunostaining with the indicated antibodies (red) and for nuclei with DAPI (4′,6-diamidino-2-phenylindole, blue) and analyzed by confocal microscopy as described in Methods or (**B**), subjected to subcellular separation into membrane and cytosolic fractions. Following centrifugation, an equal proportion of each fraction (ca. 6X more cytosol protein than membrane) were analyzed by SDS-PAGE and Western blotting. Glyceraldehyde phosphate dehydrogenase (GAPDH) is a loading control for the cytosolic fraction. (**C**) Cultured at cells were treated with or without methylβ-cyclodextrin in combination with the proteasome inhibitor MG-132 (10 µM), the lysosomal inhibitor chloroquine (40 µM), or the inhibitors alone for 90 minutes and cell extracts were prepared in lysis buffer and analyzed by SDS-PAGE and Western blotting for the proteins indicated. Shown are representative experiments.

**Figure 3:**
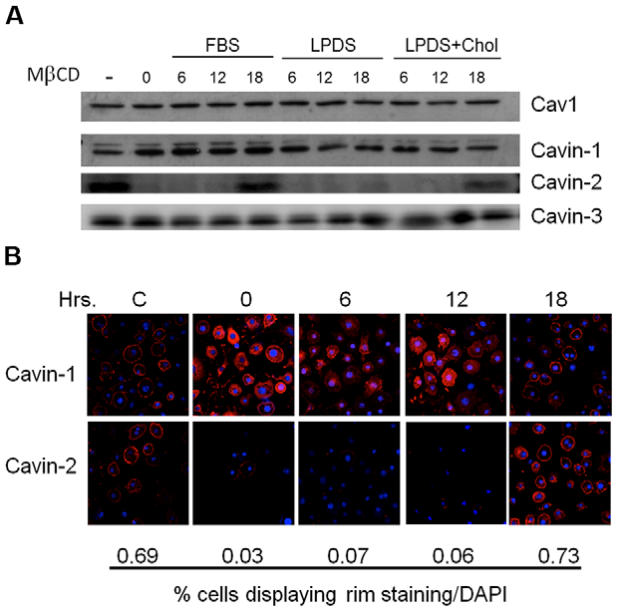
Cholesterol repletion restores cavin-2 levels, which allows return of cavin-1 to the plasma membrane. 3T3-L1 adipocytes were treated without or without methyl-β-cyclodextrin (20 mM, 90 minutes), and the medium was changed to that containing either 10% FBS, 10% FBS depleted of lipoproteins (LPDS), or, 10% FBS lipoprotein depleted serum containing cholesterol loaded cyclodextrin (25 µM cholesterol). Following incubation for the times indicated (time 0 being after MβCD removal), cell lysates were analyzed by SDS-PAGE and Western blotting (**A**) or were processed for immunofluorescence with antibodies for cavin-1 and -2 (**B**) and for nuclei (DAPI) as in prior figures. The percent cavin-2 rim staining was determined by scoring 104+/−3 cells that were also positive for DAPI staining.

**Figure 4:**
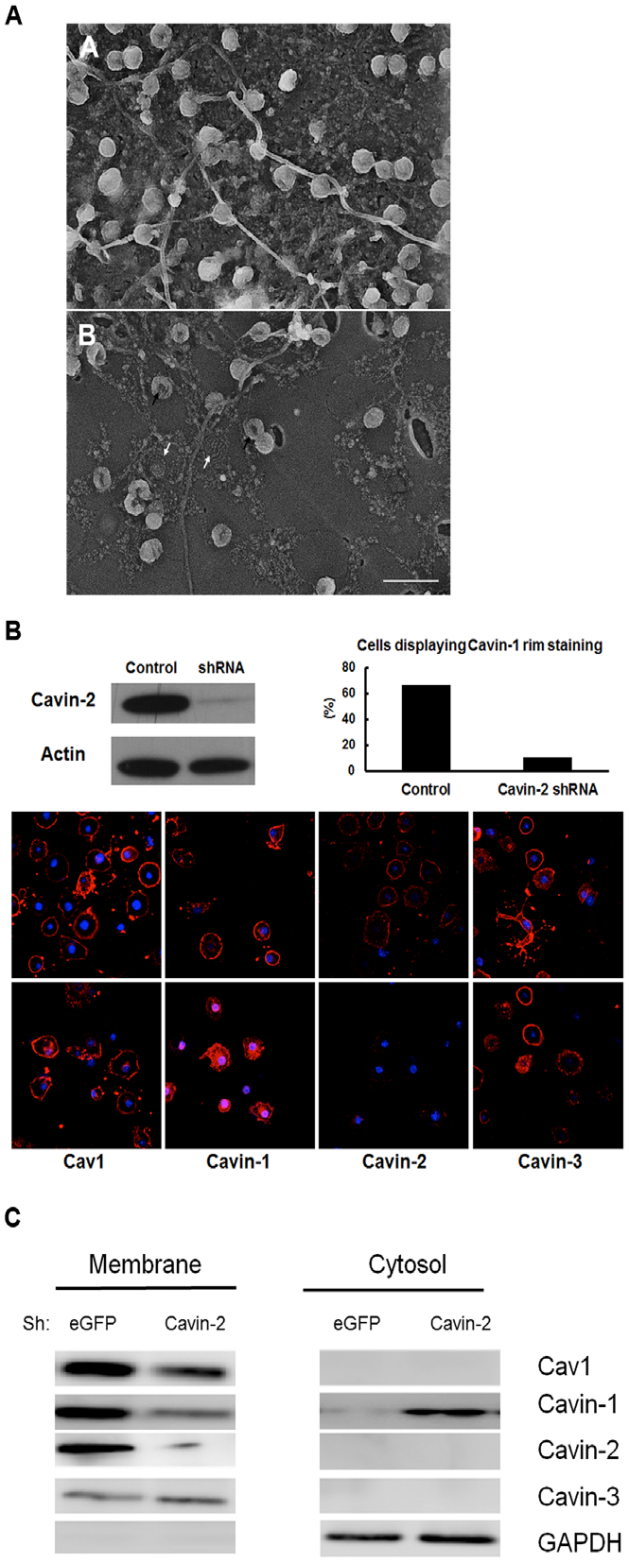
ShRNA mediated knockdown of cavin-2 causes redistribution of cavin-1 to the cytosol. 3T3-L1 adipocytes stably expressing shRNA directed against enhanced green fluorescent protein (eGFP) or cavin-2 were processed for freeze drying electron microscopy (**A**) or subjected to immunostaining (**B**) with the indicated antibodies (red) and DAPI (blue), and analyzed by confocal microscopy and quantitatively analyzed as in Figure 3 for 102 cells or (**C**) subcellular fractionation into membrane and cytosolic fractions as in Figure 2B. Following fractionation, proteins were analyzed by SDS-PAGE and Western blotting, with glyceraldehyde phosphate dehydrogenase (GAPDH) being used as a loading control for the cytosolic fraction. In Figure 4A, panel B, black arrows point to torus shaped caveolae and white arrows to flattened caveolae and the scale bar is 200 nm. In 4B, the amount of cavin-2 in whole cell lysates is shown. These are representative of 3 such experiments.

In order to understand the fate of the caveolar components upon cholesterol depletion, in Figure 2 we followed their distribution by immunofluorescence, and by SDS-PAGE after cell fractionation and exposure to inhibitors of protein degradation. As shown in Figure 2A by immunofluorescence and in 2B by cell fractionation, Cav1 and the cavins all exhibit primarily (cavin-1, 81±3%) plasma membrane (cell rim) staining in the control condition, but cavin-1 redistributes to the cytosol (83±6%, 100,000× g supernatant, see also reference 31) upon cholesterol depletion and loss of cavin-2, whereas Cav1 and cavin-3 remain associated with the PM under these conditions. The loss of cavin-2 is a result of its proteosomal degradation as is shown in Figure 2C by Western blot. The proteosomal inhibitor, MG-132, prevents loss of cavin-2, whereas the lysosomal inhibitor chloroquine (CQ) is without effect, and neither inhibitor has any effect in the absence of MβCD. We cannot rule out, however, that the punctate cavin-1 staining seen in Figure 2 (and in Figures 3 & 4), is due to its association with intracellular membranes, from which it dissociates upon centrifugation (Figure 2B).

### Cavin-2 re-expression and membrane targeting occurs upon cholesterol repletion

The recovery of cavin-2 protein expression after cholesterol depletion is dependent on cholesterol delivered by either serum lipoproteins (Figure 3A, left lanes) or by its direct addition in serum free medium (3A, right most lanes), and does not occur in the absence of a cholesterol source (3A, middle lanes) over the time frame of 12–18 hours required for caveolar recovery. The prolonged recovery is observed despite the presence of a large free cholesterol pool in the adipocyte lipid droplet [41]. Cavin-1 is re-localized to the PM only upon the re-expression and return of cavin-2 to the PM (Figure 3B). The time course of caveolae recovery data in Figure 3A and 3B is essentially identical to that shown by Hailstones et al. who showed that restoration of membrane cholesterol also required 18–24 hours [28].

### Cavin-2 silencing redistributes cavin 1 to the cytosol

Expression of shRNA is an independent and highly specific experimental approach to deplete a target protein, and as shown in Figure 4A by freeze drying EM, Figure 4B by immunofluorescence and Figure 4C by cell fractionation, shRNA directed against cavin-2 causes >95% loss of this protein and dramatically reduces caveolae number and alters cavin-1 distribution (Cont., 89±6% membrane-associated, 9±3% cytosol *versus* 11±5% membrane, 91±6% cytosol). The freeze drying EM of the intracellular face of the PM from cavin-2 deficient adipocytes after shRNA delivery (Figure 4A, panel B) shows a marked reduction in the number of caveolae. Some of the caveolae present in the cavin-2 deficient cells show a torus shape (black arrows) and others appear completely flat (white arrows) similar to what has been described upon filipin and nystatin treatments in 3T3-L1 adipocytes, respectively [37]. Figures 4B and 4C show the redistribution of cavin-1 from the PM to the cytosol upon loss of cavin-2, but Cav1 and cavin-3 remain at the cell surface under these conditions as they do with the MβCD protocols (Figure 2A & 2B). Owing to the 48 hr. incubation required for the effects of shRNA to be manifest, there is a reduction in Cav1 levels under these conditions (Figure 4C) consistent with previous cavin-2 knockdown studies [6], [32]

### Cholesterol depletion by antibiotics and statin cause loss of cavin-2 in fat cells and fibroblasts

**Figure 5:**
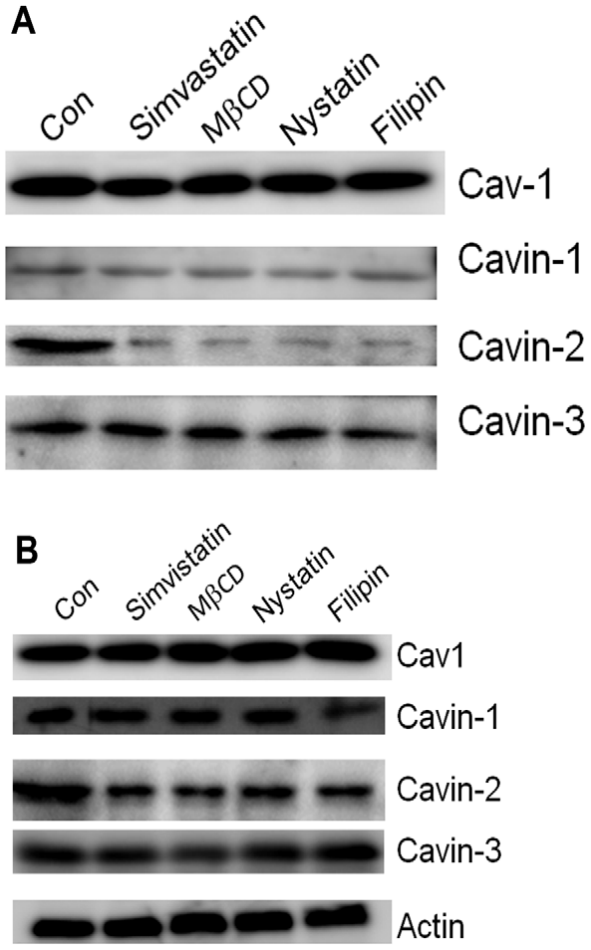
Cholesterol depletion by simvistatin and cholesterol-binding antibiotics causes loss of cavin-2 in fat cells (A) and fibroblasts (B). Adipocytes or NIH-3T3 fibroblasts were treated with or without Simvastatin (10 µM, 18 hrs) methyl-β-cyclodextrin (20 mM, 90 minutes), nystatin (76 µM, 4 hours) or filipin (7.6 µM, 4 hours). Whole cell extracts were then prepared in RIPA buffer and analyzed by SDS-PAGE and Western blotting as in prior figures.

As shown in Figure 5A, reduction in the level of cavin-2 can be achieved using 3 additional reagents targeting cholesterol sequestration and synthesis, namely overnight exposure of cells to simvastatin, an inhibitor of HMGCoA reductase [42], [43], hence cholesterol biosynthesis, and exposure to the cholesterol binding antibiotics, nystatin and filipin [43], for four hours. We also depleted cholesterol in 3T3 fibroblasts with the same agents used in Figure 5A, and as shown in Figure 5B by Western blotting, we see a significant loss of cavin-2 under these conditions with little or no change in the levels of the other cavins and Cav1. These results demonstrate that the cholesterol sensitivity of cavin-2 is not strictly a property of adipocytes.

### Cholesterol depletion in the absence of Cav1 causes redistribution of cavin 2 to the cytosol

**Figure 6:**
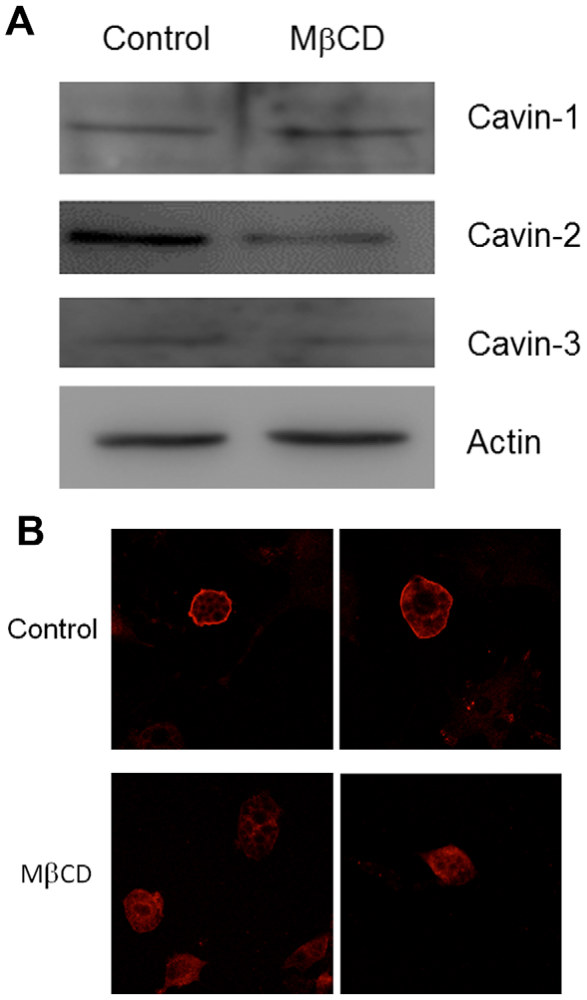
Cav1 null adipocytes lack caveolae but cavin-2 is membrane associated and redistributes to the cytosol upon cholesterol depletion. Cav1 null adipocytes were treated with or without MβCD as in previous figures and lysates (**A**) were prepared and analyzed by Western blot or (**B**) labeled with anti-cavin-2 and secondary antibody prior to analysis by confocal microscopy.

We created a permanent adipocyte cell line from Cav1 null MEFs and showed that they express all three cavins, albeit cavin-2 and cavin-3 to a lesser extent than in Cav1 positive control cells [33]. Here we show that cholesterol depletion in the Cav1 null fat cells also results in loss of cavin-2 (Figure 6A), although to a lesser degree than observed in wild type fat cells (see Figure 1B), and this occurs with no reduction cavin-1 and cavin-3 (Figure 6A). Interestingly in Cav1 null fat cells, cholesterol depletion causes cavin-2 to redistribute to the cytosol (Figure 6B) and it is degraded to a lesser extent than in Cav1 positive cells (Figure 6A compared to Figure 1B).

## Discussion

Adipocytes have a unique cyto-architecture by virtue of their large lipid droplets, the triglyceride storage depots that represent a cell-specific organelle [25], [44], and as noted in the introduction, they have particularly abundant numbers of caveolae [24]. This last property and the fact that cultured fat cells closely resemble primary adipocytes in their biological properties make them a useful cell type for probing caveolae structure and function. Here we show that cavin-2 in adipocytes behaves like a cholesterol-dependent switch necessary for caveolar structure, and the degradation of cavin-2 by the proteasome following cholesterol depletion leads to collapse of caveolae. Interestingly, whereas all three non-muscle cavins have PEST sequences (proline, glutamate, serine, threonine residues) [6], [45] potentially rendering them susceptible to proteolytic degradation [46], only cavin-2 is degraded under these conditions (Figures 1B, 2C, 5A). Although Cav1 can be ubiquinated [47] and degraded in lysosomes [47], [48], it, like cavin-1 and -3, is stable under our experimental conditions even up to 18 hours in lipid depleted serum following cholesterol extraction (Fig. 3A).

A specific role for cholesterol in the collapse of caveolae is supported by the fact that four independent reagents that affect cholesterol levels, namely MβCD, nystatin, filipin and simvastatin all lead to loss of cavin-2 and redistribution of cavin-1 from the PM to the cytosol, and these reagents act over time courses that range from 70 min. to 18 hrs. (Figures 1, 2 and 5). Cholesterol repletion is required for recovery of cavin-2 expression and return of cavin-1 to the cell surface (Figure 3). Thus, the large amount of free cholesterol present in the adipocyte lipid droplet [41], does not appear to represent a functionally dynamic pool with regard to caveolae, at least in the overnight time course of recovery (Figure 3), and this is a unique observation of our study. The existence of a large free cholesterol pool in fat cells is consistent with the ability of cholesterol to diffuse or otherwise traffic [49] from caveolae and other cell surface pools to the lipid droplet, which may serve as a long-term (>18 hrs.) buffer for total organismal cholesterol.

Prior studies of cholesterol depletion and repletion in kidney-derived cells showed that recovery of caveolae occurred over a very narrow range of membrane cholesterol content, a threshold effect [28]. Such threshold or switch-like behavior has also been shown for the cholesterol-dependent regulation of the sterol regulatory binding protein (SREBP) in the endoplasmic reticulum (ER) by the cholesterol binding protein Scap (SREBP-cleavage-activating protein), such that regulation of SREBP is achieved by changes in ER membrane cholesterol from 3–5% of the total ER cholesterol content [50]. Our current results are complementary to these prior studies and raise the question as to what is the biochemical nature of the cholesterol-dependent “switch” for caveola structure, previously assumed to be Cav1 which is known to bind cholesterol [29]. However, cavin-2 moves from the membrane to the cytosol upon cholesterol depletion in Cav1-null cells (Figure 6) so Cav1 cannot entirely explain the cholesterol sensitivity of caveolae. Cavin-1 has a so-called crac (cholesterol recognition amino acid consensus, [51]) domain (R. Epand, personal communication) as does Cav1 [52], but the presence or absence of this sequence does not necessarily rule in or out cholesterol binding [51]. A possibility is that cavin-1 & -2 participate together as the cholesterol sensitive complex, as cavin-1 does not target to the membrane in the absence of cavin-2 (Figure 3, [32]), and therefore both cavins may need to be at the cell surface for cholesterol sensitivity. Alternatively there may be an additional, as yet unknown protein(s), involved in this process.

There have been a limited number of studies addressing the mechanism of caveolae assembly because the contribution of the cavins to caveola structure has only recently been described (reviewed in [1], [2]. Transfection of cavin-1 in Cav1 expressing cells can result in formation of morphological caveolae [6], but the present results show that cavin-2 is necessary to recruit endogenous cavin-1 to the cell surface (Figures 2 and 3), as was also shown to be the case by Hansen et al. [32]. We previously showed that that all 3 cavins formed a complex at the adipocyte cell surface [6], although cavin-3 appears to be less abundant than the other cavins in this context and may be more involved in caveolar dynamics than as a structural requirement [53]. Possible cavin-3 functions aside from caveolae are consistent with the observation that cavin-3 is expressed in brain and liver where there is very little expression of the other cavins and Cav1 & 2 [6]. Hayer et al. provided evidence that a cavin complex could form in the cytosol and be recruited to the PM upon the arrival there of a Cav1 scaffold complex [54]. However, we observe cavins at the PM in Cav1 null adipocytes (Figure 6) and there may therefore be additional proteins that are required for mature caveolae formation, for example, the adaptor protein, pacsin-2 [55]. In this regard, our present studies target endogenous proteins in adipocytes where the abundant caveolae may play multiple biological roles, particularly with regard to lipid traffic and storage [3], [4], [22], and the situation my be different for gain of function experiments in other cell types that normally have few caveolae. Additional work is needed to further establish protein (and lipid) hierarchies in caveola assembly.

The adipocyte is a terminally differentiated specialized cell and it is of interest to know whether or not the cholesterol sensitivity of cavin-2 is specific to this cell type. Indeed we show that it is not, as fibroblasts also show loss of cavin-2 upon exposure to four cholesterol-perturbing reagents (Figure 5B). In this case however, there appears to be a somewhat lesser effect than in adipocytes. We have also exposed Chinese Hamster Ovary (CHO) cells to statins and they also lose cavin-2 protein. Thus, the cholesterol requirement for cavin-2 membrane association appears to be a general phenomenon. In summary, we describe a novel role for cavin-2 in sensing/responding to membrane cholesterol, probably in concert with another membrane component. Loss of cavin-2 causes collapse of caveolae and results in relocalization of cavin-1 to the cytosol.

## References

[1] Hansen CG, Nichols BJ (2010). Exploring the caves: cavins, caveolins and caveolae.. Trends Cell Biol.

[2] Bastiani M, Parton RG (2010). Caveolae at a glance.. J Cell Sci.

[3] Pilch PF, Souto RP, Liu L, Jedrychowski MP, Berg EA (2007). Cellular spelunking: exploring adipocyte caveolae.. J Lipid Res.

[4] Pilch PF, Liu L (2011). Fat caves: caveolae, lipid trafficking and lipid metabolism in adipocytes.. Trends in endocrinology and metabolism: TEM.

[5] Cohen AW, Hnasko R, Schubert W, Lisanti MP (2004). Role of caveolae and caveolins in health and disease.. Physiological reviews.

[6] Bastiani M, Liu L, Hill MM, Jedrychowski MP, Nixon SJ (2009). MURC/Cavin-4 and cavin family members form tissue-specific caveolar complexes.. J Cell Biol.

[7] Drab M, Verkade P, Elger M, Kasper M, Lohn M (2001). Loss of caveolae, vascular dysfunction, and pulmonary defects in caveolin-1 gene-disrupted mice.. Science.

[8] Razani B, Engelman JA, Wang XB, Schubert W, Zhang XL (2001). Caveolin-1 null mice are viable but show evidence of hyperproliferative and vascular abnormalities.. J Biol Chem.

[9] Zhao YY, Liu Y, Stan RV, Fan L, Gu Y (2002). Defects in caveolin-1 cause dilated cardiomyopathy and pulmonary hypertension in knockout mice.. Proc Natl Acad Sci U S A.

[10] Cohen AW, Razani B, Wang XB, Combs TP, Williams TM (2003). Caveolin-1-deficient mice show insulin resistance and defective insulin receptor protein expression in adipose tissue.. Am J Physiol Cell Physiol.

[11] Cao H, Alston L, Ruschman J, Hegele RA (2008). Heterozygous CAV1 frameshift mutations (MIM 601047) in patients with atypical partial lipodystrophy and hypertriglyceridemia.. Lipids Health Dis.

[12] Kim CA, Delepine M, Boutet E, El Mourabit H, Le Lay S (2008). Association of a homozygous nonsense caveolin-1 mutation with Berardinelli-Seip congenital lipodystrophy.. The Journal of clinical endocrinology and metabolism.

[13] Galbiati F, Engelman JA, Volonte D, Zhang XL, Minetti C (2001). Caveolin-3 null mice show a loss of caveolae, changes in the microdomain distribution of the dystrophin-glycoprotein complex, and t-tubule abnormalities.. J Biol Chem.

[14] Capozza F, Combs TP, Cohen AW, Cho YR, Park SY (2005). Caveolin-3 knockout mice show increased adiposity and whole body insulin resistance, with ligand-induced insulin receptor instability in skeletal muscle.. Am J Physiol Cell Physiol.

[15] Woodman SE, Sotgia F, Galbiati F, Minetti C, Lisanti MP (2004). Caveolinopathies: mutations in caveolin-3 cause four distinct autosomal dominant muscle diseases.. Neurology.

[16] Liu L, Brown D, McKee M, Lebrasseur NK, Yang D (2008). Deletion of Cavin/PTRF causes global loss of caveolae, dyslipidemia, and glucose intolerance.. Cell metabolism.

[17] Hayashi YK, Matsuda C, Ogawa M, Goto K, Tominaga K (2009). Human PTRF mutations cause secondary deficiency of caveolins resulting in muscular dystrophy with generalized lipodystrophy.. J Clin Invest.

[18] Dwianingsih EK, Takeshima Y, Itoh K, Yamauchi Y, Awano H (2010). A Japanese child with asymptomatic elevation of serum creatine kinase shows PTRF-CAVIN mutation matching with congenital generalized lipodystrophy type 4.. Mol Genet Metab.

[19] Rajab A, Straub V, McCann LJ, Seelow D, Varon R (2010). Fatal cardiac arrhythmia and long-QT syndrome in a new form of congenital generalized lipodystrophy with muscle rippling (CGL4) due to PTRF-CAVIN mutations.. PLoS Genet.

[20] Shastry S, Delgado MR, Dirik E, Turkmen M, Agarwal AK (2010). Congenital generalized lipodystrophy, type 4 (CGL4) associated with myopathy due to novel PTRF mutations.. Am J Med Genet A.

[21] Le Lay S, Blouin CM, Hajduch E, Dugail I (2009). Filling up adipocytes with lipids. Lessons from caveolin-1 deficiency.. Biochim Biophys Acta.

[22] Pilch PF, Meshulam T, Breen M, Liu L (2011). Caveolae and lipid trafficking in adipocytes.. Current Lipidology.

[23] Guilherme A, Virbasius JV, Puri V, Czech MP (2008). Adipocyte dysfunctions linking obesity to insulin resistance and type 2 diabetes.. Nat Rev Mol Cell Biol.

[24] Thorn H, Stenkula KG, Karlsson M, Ortegren U, Nystrom FH (2003). Cell surface orifices of caveolae and localization of caveolin to the necks of caveolae in adipocytes.. Mol Biol Cell.

[25] Murphy S, Martin S, Parton RG (2009). Lipid droplet-organelle interactions; sharing the fats.. Biochim Biophys Acta.

[26] Krause BR, Hartman AD (1984). Adipose tissue and cholesterol metabolism.. J Lipid Res.

[27] Rothberg KG, Heuser JE, Donzell WC, Ying YS, Glenney JR (1992). Caveolin, a protein component of caveolae membrane coats.. Cell.

[28] Hailstones D, Sleer LS, Parton RG, Stanley KK (1998). Regulation of caveolin and caveolae by cholesterol in MDCK cells.. J Lipid Res.

[29] Murata M, Peranen J, Schreiner R, Wieland F, Kurzchalia TV (1995). VIP21/caveolin is a cholesterol-binding protein.. Proc Natl Acad Sci U S A.

[30] Hill MM, Bastiani M, Luetterforst R, Kirkham M, Kirkham A (2008). PTRF-Cavin, a conserved cytoplasmic protein required for caveola formation and function.. Cell.

[31] Liu L, Pilch PF (2008). A critical role of cavin (polymerase I and transcript release factor) in caveolae formation and organization.. J Biol Chem.

[32] Hansen CG, Bright NA, Howard G, Nichols BJ (2009). SDPR induces membrane curvature and functions in the formation of caveolae.. Nat Cell Biol.

[33] Meshulam T, Breen MR, Liu L, Parton RG, Pilch PF (2011). Caveolins/caveolae protect adipocytes from fatty acid-mediated lipotoxicity.. Journal of lipid research.

[34] Green H, Kehinde O (1975). An established preadipose cell line and its differentiation in culture. II. Factors affecting the adipose conversion.. Cell.

[35] Meshulam T, Simard JR, Wharton J, Hamilton JA, Pilch PF (2006). Role of caveolin-1 and cholesterol in transmembrane fatty acid movement.. Biochemistry.

[36] Folch J, Lees M, Sloane Stanley GH (1957). A simple method for the isolation and purification of total lipides from animal tissues.. The Journal of biological chemistry.

[37] Ros-Baro A, Lopez-Iglesias C, Peiro S, Bellido D, Palacin M (2001). Lipid rafts are required for GLUT4 internalization in adipose cells.. Proceedings of the National Academy of Sciences of the United States of America.

[38] Gonzalez-Munoz E, Lopez-Iglesias C, Calvo M, Palacin M, Zorzano A (2009). Caveolin-1 loss of function accelerates glucose transporter 4 and insulin receptor degradation in 3T3-L1 adipocytes.. Endocrinology.

[39] Heuser JE, Anderson RG (1989). Hypertonic media inhibit receptor-mediated endocytosis by blocking clathrin-coated pit formation.. The Journal of cell biology.

[40] Parpal S, Karlsson M, Thorn H, Stralfors P (2001). Cholesterol depletion disrupts caveolae and insulin receptor signaling for metabolic control via insulin receptor substrate-1, but not for mitogen-activated protein kinase control.. The Journal of biological chemistry.

[41] Krause BR, Hartman AD (1978). Quantification of adipocyte free and esterified cholesterol using liquid gel chromatography.. Journal of lipid research.

[42] Goldstein JL, Brown MS (1990). Regulation of the mevalonate pathway.. Nature.

[43] Smart EJ, Anderson RG (2002). Alterations in membrane cholesterol that affect structure and function of caveolae.. Methods in enzymology.

[44] Bickel PE, Tansey JT, Welte MA (2009). PAT proteins, an ancient family of lipid droplet proteins that regulate cellular lipid stores.. Biochim Biophys Acta.

[45] Aboulaich N, Vainonen JP, Stralfors P, Vener AV (2004). Vectorial proteomics reveal targeting, phosphorylation and specific fragmentation of polymerase I and transcript release factor (PTRF) at the surface of caveolae in human adipocytes.. The Biochemical journal.

[46] Rechsteiner M, Rogers SW (1996). PEST sequences and regulation by proteolysis.. Trends in biochemical sciences.

[47] Hayer A, Stoeber M, Ritz D, Engel S, Meyer HH (2010). Caveolin-1 is ubiquitinated and targeted to intralumenal vesicles in endolysosomes for degradation.. The Journal of cell biology.

[48] Forbes A, Wadehra M, Mareninov S, Morales S, Shimazaki K (2007). The tetraspan protein EMP2 regulates expression of caveolin-1.. The Journal of biological chemistry.

[49] Le Lay S, Hajduch E, Lindsay MR, Le Liepvre X, Thiele C (2006). Cholesterol-induced caveolin targeting to lipid droplets in adipocytes: a role for caveolar endocytosis.. Traffic.

[50] Radhakrishnan A, Goldstein JL, McDonald JG, Brown MS (2008). Switch-like control of SREBP-2 transport triggered by small changes in ER cholesterol: a delicate balance.. Cell metabolism.

[51] Epand RM (2008). Proteins and cholesterol-rich domains.. Biochimica et biophysica acta.

[52] Epand RM, Sayer BG, Epand RF (2005). Caveolin scaffolding region and cholesterol-rich domains in membranes.. Journal of molecular biology.

[53] McMahon KA, Zajicek H, Li WP, Peyton MJ, Minna JD (2009). SRBC/cavin-3 is a caveolin adapter protein that regulates caveolae function.. EMBO J.

[54] Hayer A, Stoeber M, Bissig C, Helenius A (2010). Biogenesis of caveolae: stepwise assembly of large caveolin and cavin complexes.. Traffic.

[55] Hansen CG, Howard G, Nichols BJ (2011). Pacsin 2 is recruited to caveolae and functions in caveolar biogenesis.. Journal of cell science.

